# Metabolic support of trained immune responses in myeloid cells

**DOI:** 10.7554/eLife.108814

**Published:** 2026-06-19

**Authors:** Aitor Jarit-Cabanillas, Gillian Dunphy, Federico Virga, Jan Van den Bossche, David Sancho

**Affiliations:** 1 Immunobiology Laboratory, Centro Nacional de Investigaciones Cardiovasculares CNIC Madrid Spain; 2 Escuela de Doctorado, Universidad Autónoma de Madrid Madrid Spain; 3 Department of Molecular Cell Biology and Immunology, Amsterdam Institute for Immunology and Infectious Diseases, Amsterdam Cardiovascular Sciences, Amsterdam UMC, Vrije Universiteit Amsterdam Amsterdam Netherlands; Münster University Hospital Germany; National Institute of Immunology India

**Keywords:** trained immunity, myeloid cells, metabolism

## Abstract

Trained immunity (TI) is defined as a form of innate immune memory characterised by a long-lasting ability to develop enhanced responses to a secondary challenge, whether of the same or a different nature than the initial stimulus. This process is mediated by several established hallmarks, most prominently the existence of activating epigenetic marks and metabolic adaptations. The activating epigenetic marks prime the expression of immune-related genes and are a direct driving force behind the increased cytokine production after secondary stimulation of trained monocytes and macrophages. Training stimuli also induce specific metabolic adaptations, such as the upregulation of glycolysis and lactate production or the activation of glutaminolysis leading to fumarate accumulation, which in turn promotes epigenetic changes. However, the mechanisms linking these epigenetic and metabolic changes to a TI phenotype are varied, and not all stimuli that increase glycolysis promote training, whereas some stimuli such as lipopolysaccharide (LPS) display a non-monotonic induction of TI. In addition to metabolism directly driving epigenetic changes, early gene expression changes can also reshape cell metabolism to promote a trained phenotype. In this review we aim to separate two main types of metabolic rewiring that have not been previously uncoupled. Firstly, those primary metabolic changes occurring during the initial stimulation, which precede TI induction by altering the epigenomic landscape around inflammatory genes. Secondly, those metabolic adaptations arising later as a consequence of the first wave of epigenetic regulation, which support an enhanced functional state of macrophages.

## Introduction

According to the classical view in immunology, adaptive immune cells can generate antigen-specific long-lasting immunological memory to protect against subsequent challenges, whereas innate immune cells are unable to develop memory traits (Marshall et al., 2018; Netea et al., 2011; Netea et al., 2020). Nevertheless, some memory traits are found in organisms devoid of an adaptive immune system. Invertebrates, including earthworms, cockroaches, and corals, showed a form of innate immunological memory, exhibiting protection against reinfection, or accelerated rejection response upon subsequent exposure to the same foreign tissue in graft models (Cooper and Roch, 1986; Little et al., 2005; Kurtz and Franz, 2003). In plants, a similar process occurs whereby localised pathogenic infections can trigger systemic and nonspecific protection against reinfection, a process known as systemic acquired resistance (Fu and Dong, 2013). These findings, together with the beneficial nonspecific effects of vaccination (Aaby and Benn, 2012), challenged the classical paradigm and led to the discovery that innate immune cells that have encountered a first proinflammatory insult can develop an enhanced immune response to a second challenge of either the same or a different nature, a process called trained immunity (TI) (Netea et al., 2011; Netea et al., 2020). Additionally, innate immune cells have not only acquired memory traits with the consequence of enhancing subsequent responses, but can also display blunted secondary responses after certain primary stimuli, generally lipopolysaccharide (LPS), a process termed endotoxin tolerance (Liu et al., 2019).

TI can be induced in several mature, differentiated immune cell types, in a process known as peripheral TI (Ochando et al., 2023). The first studies of this phenomenon reported that mouse and human monocytes were able to develop innate immune memory (Quintin et al., 2012; Kleinnijenhuis et al., 2012). It has subsequently been demonstrated that TI can be induced in other innate immune cells from the myeloid lineage, such as neutrophils (Kalafati et al., 2020) and dendritic cells (Hole et al., 2019), and in lymphoid cells like natural killer (Kleinnijenhuis et al., 2014) cells and γδ T cells (Röring et al., 2024). Moreover, non-immune cells such as epithelial stem cells may also display immune memory features (Bigot et al., 2019). In vivo it was demonstrated that trained-immunity-inducing agonists can induce expansion of hematopoietic progenitors, specifically those promoting myelopoiesis, in a process termed central TI (Mitroulis et al., 2018; Kaufmann et al., 2018; Tran et al., 2025). To date, several compounds have been found to induce trained phenotypes. Among these, microbe-associated molecular patterns, such as β-glucans from the yeast cell wall (Quintin et al., 2012), bacterial flagellin (Ifrim et al., 2014), and muramyl dipeptide (Ifrim et al., 2014), induce a trained phenotype in myeloid cells. Live vaccines like Bacille Calmette-Guérin (BCG) employed against tuberculosis, and systemically disseminated microbiota that reach the bone marrow (particularly *Enterococcus faecalis*) induce TI and mediate protection against heterologous infections (Kleinnijenhuis et al., 2012; Robles-Vera et al., 2025). Moreover, mucosal administration of inactivated bacteria, such as MV130 polybacterial preparations, also leads to viral- and fungal-protective TI induction (del Fresno et al., 2021; Brandi et al., 2022). Not only microbial components but also endogenous sterile triggers of inflammation, like oxidised low-density lipoproteins (oxLDLs) (Bekkering et al., 2014; Sohrabi et al., 2019) or labile heme (Jentho et al., 2021), can induce trained phenotypes.

The diversity of TI inducers and immune cells in which TI can be exerted underlines the complexity and variety of mechanisms involved in the establishment of trained phenotypes. Moreover, the same stimuli may induce a non-linear/non-monotonic response based on the dose employed. For example, while LPS generally induces tolerance at higher concentrations (e.g. 100 ng/mL), very low doses (≤10 pg/mL) promote TI; similar dose-dependent effects have been reported for flagellin (Ifrim et al., 2014). Upon primary stimulation, TI inducers activate signalling cascades and downstream transcription factors (TFs) that promote the first-wave immune response. In addition, this signalling triggers two main processes that are required for TI induction: a profound metabolic reprogramming and the deposition of epigenetic marks on chromatin. These changes create a poised chromatin state that enables a heightened immune response to future stimuli (Netea et al., 2020; Ochando et al., 2023). Notably, despite leading to opposite functional states, tolerance is also regulated by chromatin modifications (Foster et al., 2007), indicating a shared cross-cutting mechanism of innate immune ‘memory’.

Epigenetics is defined as the heritable changes in gene expression that do not alter the DNA sequence, which are mediated by histone post-translational modifications and changes in DNA methylation (Allis and Jenuwein, 2016). Epigenetic reprogramming is believed to be one of the main molecular processes driving the enhanced cytokine production upon secondary stimulation in TI. This aligns with the similar concept of ‘transcriptional memory’ (Ostuni et al., 2013; Glass and Natoli, 2016). The metabolic and epigenetic rewiring that occurs during TI induction is highly interconnected (Martínez-Reyes and Chandel, 2020). It has been demonstrated that metabolic rewiring is a prerequisite for the epigenetic reprogramming that drives enhanced production of proinflammatory cytokines after secondary stimulation. In parallel, epigenetic rewiring drives metabolic changes that are required for macrophage function.

The timing of metabolic and epigenetic crosstalk in TI is crucial, as this interplay is both rapid and sustained (Arts et al., 2016b; van der Heijden et al., 2018). Shortly after the primary stimulation, a profound epigenetic reprogramming occurs. This epigenetic rewiring can influence the expression of metabolic enzymes, thereby modifying the metabolic adaptations required for macrophage immune responses. Nevertheless, the epigenetic changes are not completely fixed within the first hours after the primary stimulation (Novakovic et al., 2016), and changes in metabolism, such as increased glycolysis and production of intermediates like lactate, acetyl-CoA, and fumarate, can influence the activity of epigenetic enzymes (e.g. histone acetyltransferases [HATs] and demethylases). These metabolic changes can modulate epigenetic modifications in a period of time that spans from hours after the primary stimulation to even days after the initial trigger. In this review, we aim to highlight the epigenetic-metabolic crosstalk in TI in monocytes and macrophages, distinguishing the primary metabolic changes that influence early epigenetic adaptations driving TI induction from secondary metabolic adaptations that are a consequence of this epigenetic rewiring. Finally, we overview methodological techniques to explore the metabolic reprogramming during TI induction.

## Primary metabolic adaptations orchestrate epigenetics

Cell metabolism can be seen not only as a means of macromolecule and energy synthesis but also as a way to communicate external cues to elicit specific functional responses. One way that this communication occurs is through changes in metabolite levels that can regulate epigenetic modifications (Martínez-Reyes and Chandel, 2020; Etchegaray and Mostoslavsky, 2016; Britt et al., 2020). Cellular metabolites and especially mitochondrial TCA cycle metabolites play a key role in this communication, acting as substrates or cofactors for enzymes that affect DNA methylation status and modification of histones. Thus, by retrograde signalling, cytosolic and mitochondrial metabolism influences the expression of nuclear-encoded genes by modulating epigenetic modifications (Martínez-Reyes and Chandel, 2020; Chandel, 2015). Another demonstrated mechanism of mitochondrial-nuclear communication is the nuclear translocation of mitochondrial proteins (Monaghan and Whitmarsh, 2015). While the cellular compartmentalisation of metabolites and proteins has not been addressed in the context of TI, this may be an important factor in metabolic regulation of transcription. Epigenetic rewiring, which increases the accessibility of innate immune genes and poises them for transcription, underlies the enhanced production of proinflammatory cytokines in TI. High-throughput sequencing technologies (Assay for Transposase-Accessible Chromatin using sequencing [ATAC-seq], ChIP-seq, and CUT&TAG) have revealed that TI-inducing stimuli trigger a genome-wide expansion of accessible chromatin (Novakovic et al., 2016; Saeed et al., 2014; Cai et al., 2025). For instance, β-glucan stimulation leads to a global epigenetic change at distal regulatory elements (enhancers) and promoters, which remain poised even after the initial stimulus is removed (van der Heijden et al., 2018; Novakovic et al., 2016; Saeed et al., 2014). In this context, multiple metabolic pathways that contribute to the initiation and/or maintenance of the epigenetic rewiring that assists the trained phenotype have been described.

### Histone acylation: acetyl-CoA and lactate

**Figure 1.**
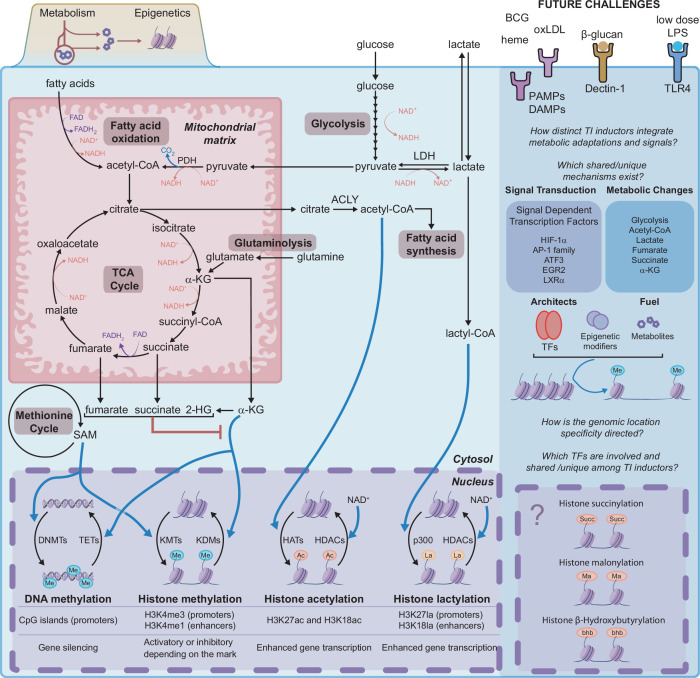
Cellular metabolites control epigenetic modifications during trained immunity (TI) induction. Main metabolic pathways, depicted with a maroon background, involved in TI induction through epigenetic modifications in both the mitochondria (red background) and the cytosol (blue background). Metabolic directions of metabolites are represented with black arrows, while blue arrows show the crosstalk between specific metabolites and epigenetic modifications. TCA cycle metabolites like α-ketoglutarate (αKG), succinate, and fumarate affect enzymatic activity of histone and DNA demethylases. S-adenosylmethionine (SAM), produced in the methionine cycle, promotes histone and DNA methylation. Citrate-derived acetyl-CoA is required for histone acetylation, while NAD^+^ is a cofactor required for sirtuin histone deacetylases (HDACs). Lactate promotes histone lactylation, which works in a similar way as histone acetylation, loosening chromatin structure and activating gene transcription. Epigenetic modifications are shown in the nucleus (purple background). DNA methyltransferases (DNMTs) and ten-eleven-translocation (TET) proteins are involved in DNA methylation and demethylation, respectively. Histone acetyltransferases (HATs), like p300, and HDACs, trigger histone acetylation/lactylation and deacetylation/delactylation, respectively. Histone lysine methyltransferases (KMTs) and histone lysine demethylases (KDMs) are involved in methyl transfer from SAM to histone lysine residues and its removal, respectively. LDH, lactate dehydrogenase; ACLY, ATP citrate lyase; PDH, pyruvate dehydrogenase; NAD^+^, nicotinamide adenine dinucleotide; NADH, nicotinamide adenine dinucleotide reduced; 2-HG, 2-hydroxyglutarate; TF, transcription factors; SAM, S-adenosylmethionine; PAMP, pathogen-associated molecular pattern; DAMP, damage-associated molecular pattern.

One of the main epigenetic modifications described to contribute to the trained phenotype is histone acylation, with histone acetylation being one of the first and most commonly studied to date (Novakovic et al., 2016; Saeed et al., 2014; Cheng et al., 2014). HATs, assisted by transcription factors (TFs) that help to direct its activity, promote acetylation using acetyl-CoA as a substrate, generally enhancing transcriptional activity by generating H3K9ac and H3K27ac, which neutralise the positive charges on lysine residues, leading to a more open chromatin structure (Figure 1). In turn, acetyl marks also recruit TFs and chromatin remodelling complexes to activate transcription (Allis and Jenuwein, 2016). TI inducers such as β-glucan (Arts et al., 2016b; Novakovic et al., 2016; Saeed et al., 2014; Cheng et al., 2014), heme (Jentho et al., 2021), and BCG (Arts et al., 2018; Kaufmann et al., 2018; Vierboom et al., 2021), among others, promote the deposition of H3K27ac, which specifically marks active promoters and enhancers, triggering a massive reconfiguration of the epigenome. H3K27ac was initially described to be one of the key and highly dynamic marks upon β-glucan stimulation of human monocytes, more often at distal regions compared to regions nearby the transcription start site (Novakovic et al., 2016; Saeed et al., 2014). The initial deposition of histone marks is assisted by the signalling pathways elicited by the primary stimulation. TF motif analyses revealed that motifs for Basic Leucine Zipper (bZIP) subfamily members, specifically Activating Transcription Factor 1 (ATF1), ATF7, cAMP Responsive Element Binding Protein 3 (CREB3), and Jun dimerisation protein 2 (JDP2), along with the glucocorticoid receptor (NR3C1), were significantly enriched at dynamic H3K27ac sites induced by β-glucan (Saeed et al., 2014). Notably, tolerised macrophages can be, at least partially, rescued by β-glucan treatment that can induce most of the H3K27 deposition at the regions silent in tolerised macrophages (Novakovic et al., 2016). When comparing β-glucan and LPS-treated monocytes, β-glucan-induced enhancers were specifically enriched for TF motifs such as Early Growth Response 2 (EGR2), a downstream element of the Dectin-1 pathway. These EGR2-linked regions were associated with lipid metabolism and lysosomal genes. This global H3K27ac reprogramming across the genome suggests that TI does not merely switch on a few genes, but rather lowers the kinetic barrier for an entire network of thousands of loci involved in distinct processes, including cytokine production, leukocyte activation, phagocytosis, and cellular metabolism.

These dynamic changes in histone acetylation are associated with accumulation of acetyl-CoA during TI induction by various stimuli (Arts et al., 2016b; Sohrabi et al., 2020; Findeisen et al., 2022). Acetyl-CoA can be generated from several metabolic pathways (Figure 1). For instance, increased glycolysis results in the production of pyruvate, which is transported into the mitochondria and converted to acetyl-CoA by the pyruvate dehydrogenase complex. Moreover, fatty acid β-oxidation breaks down fatty acids into acetyl-CoA molecules in the mitochondrial matrix. Nevertheless, for acetyl-CoA to impact histone acetylation, these molecules must act in the cytosolic/nuclear fraction (Wellen et al., 2009). Acetyl-CoA-derived citrate within the TCA cycle can be exported to the cytosol and cleaved by the enzyme ATP citrate lyase (ACLY) to generate acetyl-CoA as a precursor in mevalonate/cholesterol biosynthesis and for the acetylation of histones and other proteins (Wellen et al., 2009; Figure 1). Trained immune cells have been found to upregulate several of the pathways that lead to acetyl-CoA accumulation, such as glycolysis (Cheng et al., 2014; Arts et al., 2016a; Keating et al., 2020a), fatty acid oxidation (Groh et al., 2021), and fatty acid synthesis (FAS) (Arts et al., 2016b). This suggests that the upregulation of these metabolic pathways could contribute to the build-up of acetyl-CoA and histone acetylation during TI induction.

Several studies have also found evidence directly linking acetyl-CoA accumulation upon training induction to histone acetylation at relevant genes for innate immune function. In the case of primary oxLDL stimulation TI, the activity of Liver X Receptor (LXR) increased levels of acetyl-CoA. This accumulation was accompanied by increased expression of *ACLY*, which was reduced upon treatment with an LXR antagonist, correlating with heightened cytokine responses to secondary stimulation (Findeisen et al., 2022). In addition, the increased H3K27ac marks at *TNF* and *IL6* promoters induced by oxLDL stimulation were reduced by pre-treatment with LXR antagonists, indicating that oxLDL-induced TI is partially mediated by LXR activity, increased acetyl-CoA, and H3K27ac deposition at the promoters of proinflammatory cytokines (Findeisen et al., 2022). In line with this study, direct LXR activation alone induced a trained phenotype in human monocytes by increasing acetyl-CoA and deposition of H3K27ac at the promoters of *TNF* and *IL6* (Sohrabi et al., 2020). In addition, mevalonate training was accompanied by global changes in H3K27ac, including inflammatory and non-inflammatory pathways, as measured by whole-genome epigenetic analysis (Bekkering et al., 2018). Nevertheless, in this study, the accumulation of acetyl-CoA was not studied, and a causative role between H3K27ac and acetyl-CoA through mevalonate cannot be affirmed. Instead, the study indicates that mevalonate training induces global H3K27ac changes via the IGF1-R/mTOR axis, modulating key inflammatory pathways. In the case of β-glucan-induced TI, one study suggested that the accumulation of acetyl-CoA and increased histone acetylation observed after stimulation is primarily driven by lactate rather than glucose (Cai et al., 2025). Lactate is taken up by the cells and converted to acetyl-CoA through a pathway involving the enzymes lactate dehydrogenase (LDH), pyruvate dehydrogenase, and ACLY. Inhibiting any of these enzymes blocked the β-glucan-induced production of acetyl-CoA, and isotope tracing confirmed that lactate was the main carbon source for this process. Interestingly, while blocking ACLY reduced histone acetylation at specific sites (such as H3K27ac and H3K18ac), it did not affect the production of key inflammatory cytokines (IL-6 and TNF), nor did it impact bacterial clearance, a role that was more ascribed to histone lactylation (Cai et al., 2025). Moreover, acetyl-CoA-mediated histone acetylation has not only been reported to affect innate immune genes, but it can also promote upregulation of glycolytic genes such as hexokinase and LDH (Wellen et al., 2009), which could in turn affect TI induction (Cheng et al., 2014).

Nevertheless, while the correlation between acetyl-CoA accumulation and histone acetylation is well established, a significant knowledge gap remains regarding the direct mechanistic coupling of specific metabolic fluxes to global epigenetic reprogramming. On one hand, the epigenetic landscape of TI has been extensively mapped across various inductors, and metabolic studies have demonstrated the necessity of certain pathways for TI induction (Novakovic et al., 2016; Saeed et al., 2014; Arts et al., 2016b; Cheng et al., 2014; Keating et al., 2020b). Yet, these studies often rely on measuring histone modifications at a few specific loci (e.g. *TNF* or *IL6* promoters) rather than assessing how specific metabolic disruptions, such as the inhibition of ACLY (Cai et al., 2025), alter the global deposition and maintenance of these marks. Integrating precise metabolic manipulations with unbiased, genome-wide profiling represents an exciting opportunity to move beyond correlative observations and decipher the systemic contribution of metabolites to the trained phenotype.

The recently identified modification, histone lactylation (Kla), provides a compelling example of how a specific metabolite fuels broader genomic impact over time. In this process similar to acetylation, lactyl-CoA derived from lactate is covalently attached to lysine residues on histone proteins via the acetyltransferase p300 (Zhang et al., 2019; Figure 1). The addition of lactyl groups to histones loosens chromatin structure and promotes the expression of specific genes, such as those involved in wound healing and tissue repair in macrophages (Zhang et al., 2019; De Leo et al., 2024; Desgeorges et al., 2024). Histone lactylation connects cellular metabolic activity, particularly lactate generation from glycolysis-derived pyruvate, to the control of gene expression. Given the common signature of increased glycolysis and lactate production after stimulation with several TI inducers (Cheng et al., 2014; Arts et al., 2016a; Keating et al., 2020a), it was proposed that histone lactylation could have a role in the epigenetic rewiring occurring during TI induction (Ziogas et al., 2025). Human monocytes from individuals vaccinated with BCG showed long-term H3K18 lactylation, displaying 4791 dynamic peaks upon BCG across the genome, predominantly at distal enhancer regions rather than promoters, enhancing transcription (Ziogas et al., 2025). A relevant example was the deposition of H3K18la in regulatory regions of the *IL1B* gene, including its enhancer and promoter, and also long-range contact sites and CTCF-binding sites, suggesting a potential involvement in modifying gene architecture and regulating IL-1β expression in trained macrophages (Ziogas et al., 2025). In addition to this important locus, and compared with LPS-tolerised macrophages, pathway analysis of peaks specific to BCG-trained macrophages revealed links to the endosomal ESCRT-III complex and NOD2 signalling. Inhibition of glycolysis or lactate production diminished the deposition of H3K18la and also TI induction, which was also suppressed by inhibitors of the HAT p300 (Ziogas et al., 2025). Similar to BCG, β-glucan stimulation of human monocytes led to global enhanced histone lactylation, specifically H3K27la in an LDHA-dependent manner, which resembled accessibility data by ATAC-seq. Immunoblot analyses showed that global histone lactylation levels were more elevated than global acetylation levels following β-glucan stimulation of monocytes (Cai et al., 2025). In addition to this widespread epigenetic change, H3K27la peaks were highly enriched at the promoters of genes associated with cell adhesion and innate immune response, such as *TNF*, *IL6*, *PSGL,* and *ICAM1*, which contributed to trained phenotypes (Cai et al., 2025; Figure 1). This histone lactylation mark was more associated with promoter regions than enhancer regions, as was the case for H3K18la, suggesting that different lactylation marks could occur preferentially at different genomic locations depending on the TI inducer (Cai et al., 2025; Ziogas et al., 2025). Beside the effect of lactate on histone lactylation, lactate can also have a profound effect on immune cell metabolism and function (Cappellesso et al., 2024). In β-glucan-treated macrophages, lactic acid fuels the TCA cycle to promote the production of citrate (Cai et al., 2025). The lactic acid-derived citrate is used as a primary source for H3K27 acetylation. In a context of LPS primary stimulation, which decreases global H3K27ac levels, lactate restores global H3K27ac while having no effect on other histone marks such as H3K28ac, H3K24ac, and H3K8ac, as observed by immunoblots. This study reported that H3K27ac driven by lactate promoted the expression of genes involved in chromatin regulation, such as the TF NR4A1 which, in turn, reduces the expression of inflammatory genes by altering chromatin accessibility. In this way, lactic acid can induce a form of long-term immunosuppression, named ‘trained immunosuppression’ (Shi et al., 2024). These studies highlight the close crosstalk between metabolic and epigenetic changes that together regulate innate immune memory.

Nevertheless, these studies leave several open questions. Mechanistically, it was identified that acetylation and lactylation operate on different timescales (Zhang et al., 2019). Histone acetylation acts as a rapid response, peaking within 3–6 hr to drive early proinflammatory genes, but these levels then decrease or slow down during the later stages of the response. In contrast, histone lactylation follows a slower trajectory, steadily increases over 24 hr to trigger homeostatic and M2-like genes, such as *Arg1*, which promotes wound healing during the late phase of infection (Zhang et al., 2019). This dual nature of lactylation acting as a ‘lactate clock’ to terminate initial inflammation in acute settings while functioning as a persistent memory mark that poises pro-inflammatory loci in TI raises a fundamental question. Analysing the specific contributions of acetylation and lactylation in long-term reprogramming is essential, especially given their distinct kinetics and that their actions may be context-dependent and stimulus-dependent rather than phenotypically fixed.

The fact that histone lactylation can potentiate proinflammatory genes during TI, while promoting homeostatic or immunosuppressive genes in other contexts, suggests a dual role. This indicates that the action of lactate and by extension the accumulation of acetate derived from lactate for histone acetylation do not seem to control the phenotypic polarisation that they exert, but they act as merely substrate reservoirs. According to this model, the metabolite environment provides the necessary fuel, while signalling cascades and the activation of specific signal-dependent TFs, such as the PERK-ATF4 axis in tumour-associated macrophages or AP-1 and ATF3 in BCG-trained cells, orchestrate the genomic specification, directing the marks to where they are functionally needed (De Leo et al., 2024; Ziogas et al., 2025). Additionally, the discovery of ‘trained immunosuppression’, where lactic acid drives a specific H3K27ac-NR4A1 axis to promote stable tolerance (Shi et al., 2024), suggests a need to investigate whether lactylation also poises homeostatic programmes in TI to balance the enhanced secondary response and prevent excessive tissue damage.

Histone deacetylases (HDACs) catalyse the removal of acetyl groups from histone tails, increasing the positive charge of histones leading to a more compact chromatin structure. This reduces accessibility for TFs and RNA polymerase, thus suppressing gene expression. However, these HDACs are not acetylation-specific and have been shown to remove other histone acylations, such as histone lactylation and succinylation (Moreno-Yruela et al., 2022; Li et al., 2023). Class III HDACs are composed of sirtuins, which catalyse the removal of acetyl groups in an NAD^+^-dependent manner (Xu et al., 2024; Figure 1). NAD^+^ and NADH are central redox cofactors that receive or donate electrons, participating in a wide range of metabolic pathways. The balance between this redox pair affects immunomodulatory functions and immune responses (Zheng et al., 2022; Quinn et al., 2020; Minhas et al., 2019). Regarding TI induction, it was previously described that an increase in the NAD^+^/NADH ratio was detected in human monocytes 3 and 6 days, but not 1 day, after β-glucan stimulation (Cheng et al., 2014), suggesting that NAD^+^ biology could impact trained responses. One of the pathways that contribute to NAD^+^ generation is the salvage pathway, which utilises enzymes like nicotinamide phosphoribosyltransferase (NAMPT) to convert nicotinamide to nicotinamide mononucleotide (NMN), a precursor to NAD^+^. The NAD^+^ salvage pathway contributes to macrophage inflammatory activity and primary macrophage responses (Cameron et al., 2019; Venter et al., 2014). Moreover, stimulation of microglia with B cell-activating factor to induce a proinflammatory memory-like response increased the NAD^+^/NADH ratio of microglia after 5 days of stimulation (Wang et al., 2021). Nevertheless, mechanistic experiments are needed to clarify how NAD^+^ contributes to the early changes in histone acetylation and their role in TI induction.

Finally, it is important to note that while histone acetylation and lactylation are more widely studied, the contribution of other histone acylations such as histone succinylation, malonylation, or β-hydroxybutyrylation may also play a role in TI (Baldensperger and Glomb, 2021; Xie et al., 2016). However, new tools will be required to decipher their specific roles.

### Histone methylation: glutaminolysis, fumarate, succinate, and itaconate

Histone methylation is a key epigenetic modification that consists of the transfer of methyl groups to specific lysine or arginine residues on histone tails. This process is catalysed by histone methyltransferases, such as the SET-domain-containing enzymes for lysine residues (Etchegaray and Mostoslavsky, 2016; Black et al., 2012; Rea et al., 2000). S-adenosylmethionine (SAM) is the universal methyl donor for histone methyltransferases. Depending on the residue and degree of methylation (mono-, di-, or trimethylation), this modification can either activate or repress gene transcription, emphasising the functional diversity of these marks. For instance, trimethylation of histone H3 at lysine 4 (H3K4me3) is generally linked to gene activation, whereas trimethylation of H3 at lysine 9 or 27 (H3K9me3, H3K27me3) is associated with transcriptional repression (van der Heijden et al., 2018; Etchegaray and Mostoslavsky, 2016; Black et al., 2012). Moreover, the degree of H3K4 methylation serves as a chromatin signature to distinguish between enhancers and promoters. H3K4me1 is the hallmark of enhancers, while H3K4me3 is a mark of active or poised promoters (van der Heijden et al., 2018; Heintzman et al., 2007). H3K4me2 is often considered an intermediate bridge mark that has not been explored in TI studies. In the context of TI, these distinctions are crucial because they define whether a gene is actively transcribed or primed at proximal or regulatory sites for a future response (Ostuni et al., 2013; van der Heijden et al., 2018; Novakovic et al., 2016). Conversely, histone demethylases such as the Jumonji C (JmjC) domain-containing family remove these methylation marks, thus inversely regulating gene expression (Cloos et al., 2008).

TI inducers have been shown to promote a profound, genome-wide reconfiguration of histone methylation (Quintin et al., 2012; van der Heijden et al., 2018; Saeed et al., 2014; Saeed et al., 2014). First, it was demonstrated that β-glucan stimulation promoted global dynamic changes of H3K4me3 in monocytes, including genes associated with the immune response, immune receptors, and atherosclerosis, many of which were detected 24 hr post stimulation and maintained for at least a week (Quintin et al., 2012; Bekkering et al., 2014). Saeed et al. performed a more profound analysis of global H3K4me3 and H3K4me1 marks upon β-glucan stimulation. They found that around 500 promoters concurrently gained H3K27ac and H3K4me3, suggesting parallel activation of promoters via distinct histone marks during TI induction (Saeed et al., 2014). Moreover, this study provided the first analysis of the dynamic changes in H3K4me1 at distal enhancers upon TI (Saeed et al., 2014), associating this particular observation with the latent or de novo enhancer generation in macrophages, which primes for subsequent immune responses (Ostuni et al., 2013; Kaikkonen et al., 2013). BCG vaccination also promoted changes in H3K4me3 and H3K4me1. Interestingly, persistent H3K4me3 enrichment was observed at the promoters of *TNF*, *IL6*, and *TLR4*, remaining detectable for 3 months to 1 year after vaccination in humans (Kaufmann et al., 2018). BCG vaccination also induced a functional reprogramming of mature neutrophils 3 months after vaccination, including widespread changes in H3K4me3 at several promoter regions (Moorlag et al., 2020b). The integration of these histone modifications suggests a hierarchical model of training. While H3K4me1 establishes a ‘latent’ enhancer landscape, the recruitment of H3K4me3 and H3K27ac provides the transcriptional ‘trigger’. This process is orchestrated by the balance of metabolites that act as either cofactors or competitive inhibitors for epigenetic enzymes, ensuring that the metabolic state of the cell is directly translated into genomic accessibility.

Many epigenetic enzymes such as TET1–3 hydroxylases with a role in DNA demethylation and the Jumonji C domain-containing lysine demethylases (KDM2–7), which mediate histone demethylation, are 2-oxoglutarate-dependent dioxygenases (2-OGDDs) (Klose et al., 2006; Scourzic et al., 2015). The activity of these enzymes depends on the ratio between their cofactor, α-ketoglutarate (αKG), and other antagonistic metabolites that compete for binding, such as succinate, fumarate, and 2-hydroxyglutarate (Xiao et al., 2012; Xu et al., 2011; Figure 1). In macrophages, αKG contributes to endotoxin tolerance by Jmjd3-dependent epigenetic modifications, and an increase in the succinate to αKG ratio was shown to enhance proinflammatory responses (Liu et al., 2017; Seim et al., 2019). Metabolomic studies in human monocytes treated with β-glucan showed an upregulation of TCA cycle metabolites such as succinate, fumarate, and malate (Arts et al., 2016b). This accumulation was consistent with induction of glutaminolysis, a metabolic pathway in which glutamine is converted into glutamate and subsequently into αKG, in order to replenish the TCA cycle (Yoo et al., 2020; Figure 1). Inhibition of glutaminolysis with BPTES reduces the expression of activating epigenetic histone marks like H3K4me3 and the enhanced production of proinflammatory cytokines induced by β-glucan training (Arts et al., 2016b). Inhibition of glutaminolysis also suppressed oxLDL-induced TI in human monocytes, resulting in decreased production of proinflammatory cytokines, which was associated with reduced glycolytic activity and OXPHOS (Scarpa et al., 2025). Additionally, this study identified associations between specific single-nucleotide polymorphisms in glutaminolysis-related genes and ex vivo cytokine production in oxLDL-trained human monocytes (Scarpa et al., 2025). BCG stimulation of human monocytes resulted in elevated levels of intracellular glutamate and malate, but not fumarate (Arts et al., 2016a). Moreover, it was described that metabolites associated with glutamine metabolism and with the TCA cycle, such as succinate and malate, were significantly enriched in individuals with robust TI responses after BCG vaccination (Koeken et al., 2022). Bone marrow-derived macrophages trained with the TLR4 agonist monophosphoryl lipid A showed a break at the enzyme isocitrate dehydrogenase (IDH) in the TCA cycle, characteristic of activated macrophages (Jha et al., 2015; Mills et al., 2016), and a reestablished flux at αKG via glutamine anaplerosis (Fensterheim et al., 2018). This indicates that elevation of TCA cycle metabolites driven by glutamine anaplerosis could contribute to TI induction.

As another competitor molecule for αKG-dependent 2-OGDDs, fumarate also inhibits histone and DNA demethylases, resulting in gene expression changes (Xiao et al., 2012; Sciacovelli et al., 2016). In the case of TI, it was shown that fumarate inhibited the activity of the histone demethylase KDM5, promoting the maintenance of the activating histone mark H3K4me3 on the promoters of TNF and IL6 (Arts et al., 2016b). Either β-glucan stimulation or the sole addition of fumarate were able to decrease KDM5 activity, enabling the deposition of H3K4me3. In agreement with fumarate promoting TI by inhibiting histone demethylases, αKG administration rescued the drop in KDM5 activity and reduced the TNF produced in trained monocytes (Arts et al., 2016b). In line with this, it was described that fumarate itself was able to induce a trained phenotype in human monocytes, promoting epigenetic changes mostly in genomic regions that were also similarly regulated by β-glucan (Arts et al., 2016b). Fumarate training alone induces 124 dynamic H3K4me3 regions and 332 dynamic H3K27ac regions at several genomic locations across the genome. These fumarate-induced changes represent a specific subset, recapitulating a small fraction of the total trained epigenome. However, 95% of the genomic regions regulated by fumarate were also regulated by β-glucan, confirming it as a key mediator of the broader response. This does not rule out that fumarate could also be involved in the deposition of histone marks upon β-glucan that do not appear in the set of dynamic regions modulated by fumarate alone. To test this, specific depletion of certain metabolites upon β-glucan treatment and downstream epigenetic analyses should be performed.

While succinate would also inhibit histone demethylase activity in a similar manner to fumarate, potentially promoting trained responses, its contribution to the epigenetic reprogramming during TI induction has not been explored. However, in the context of immune tolerance, succinate and itaconate were shown to promote DNA hypermethylation and inhibit inflammatory cytokine induction (Longlax et al., 2024). Therefore, the interplay of DNA and histone methylation by altered metabolite levels requires more investigation to understand how these processes are balanced to support TI or tolerance phenotypes. In addition to this, succinate also inhibits prolyl hydroxylases, promoting HIF-1α stabilisation, which in turn promotes the production of proinflammatory cytokines such as IL-1β (Tannahill et al., 2013). Succinate can also signal through its cell-surface receptor SUCNR1 (GPR91), further amplifying immune responses and cytokine production (Littlewood-Evans et al., 2016). While the effect of succinate in primary innate immune responses has been explored, further studies are required to understand the role, if any, of succinate accumulation during TI.

Another metabolite with important immunomodulatory properties that could modulate TI induction is itaconate. Itaconate is produced from the TCA cycle metabolite *cis*-aconitate in activated proinflammatory macrophages upon upregulation of immune response gene 1 (*Irg1*) (Jha et al., 2015; Mills et al., 2018; Michelucci et al., 2013). Due to its similar structure to succinate, itaconate can bind to the active site of succinate dehydrogenase (SDH), acting as a competitive inhibitor, producing a break in the TCA cycle and leading to the accumulation of succinate and modulation of mitochondrial metabolism (Cordes et al., 2016). This inhibition is a key mechanism by which itaconate exerts its anti-inflammatory and antioxidant effects, including the activation of TFs such as Nrf2 and suppression of the NLRP3 inflammasome (Mills et al., 2018; Hooftman et al., 2020). The relationship between itaconate and TI is complex. Itaconate production is associated with immune tolerance, and its accumulation can dampen inflammatory responses and inhibit the development of TI in monocytes (Domínguez-Andrés et al., 2019). The addition of dimethyl itaconate, a cell-permeable derivative of itaconate, before β-glucan stimulation dampened key TI signatures. Specifically, this intervention reduced the enhanced cytokine production upon restimulation and the increased lactate release characteristic of trained monocytes (Domínguez-Andrés et al., 2019). In turn, β-glucan suppressed the increased expression of *Irg1* in LPS-tolerised monocytes, preserving SDH activity and TCA cycle integrity to enhance innate immune responses (Domínguez-Andrés et al., 2019). A potential mechanism explaining the contribution of SDH activity to TI induction would be the generation of fumarate, but further studies are required to understand the interplay between itaconate, succinate, and fumarate in the induction of TI and the potential contribution to histone methylation. Furthermore, in the absence of other stimuli, dimethyl itaconate alone was able to induce long-term transcriptional, metabolic, and epigenetic changes characteristic of TI. In addition, itaconate levels in human plasma correlated with enhanced trained phenotype ex vivo (Ferreira et al., 2023b). This demonstrates that itaconate and its derivatives can have both anti-inflammatory and pro-inflammatory effects depending on the cellular context and timing (Domínguez-Andrés et al., 2019; Ferreira et al., 2023b).

A central question in TI remains how metabolically driven post-translational modifications (PTMs) achieve genomic specificity. While metabolic shifts provide the necessary substrate reservoirs, the ‘fuel’, they lack the intrinsic ability to recognise specific DNA sequences. Instead, a hierarchical model is emerging whereby signal-dependent TFs may act as the ‘architects’ of the trained epigenome. In this model, innate stimuli activate signalling cascades that mobilise TFs to specific distal enhancers and promoters. These TFs then serve as scaffolds, recruiting epigenetic enzymes (such as HATs or histone methyltransferases) and ‘erasers’ (such as KDMs) to specific loci. For example, β-glucan-induced enhancers are heavily enriched for EGR2 motifs, which direct H3K27ac deposition towards genes governing lipid metabolism and lysosomal function (Novakovic et al., 2016). Ziogas et al. confirmed that the deposition of H3K18la does not just land randomly, but the genomic regions showing deposition of this histone marks were significantly enriched for motifs of the AP-1 family and ATF3, suggesting a model in which lactate is employed as fuel and TFs as potential scaffolds that recruited the machinery for histone lactylation at specific genomic sites (Ziogas et al., 2025). However, this ‘TF-anchored’ recruitment model does not fully explain how the exogenously supplied metabolites, such as fumarate, can recapitulate specific subsets of the trained epigenome in the absence of a primary PRR stimulus. This raises the possibility that yet-to-be-identified metabolic stress sensors or non-canonical signalling properties of TCA cycle intermediates may bridge the gap between metabolite abundance and site-specific recruitment. Moreover, an additional and largely unexplored mechanism may involve the direct tethering of metabolic enzymes to chromatin, whereby their nuclear translocation generates locally high concentrations of substrates directly at the potential specific sites. Ultimately, understanding how these histone changes integrate to establish TI will require moving beyond correlative studies. Future research must integrate genome-wide histone PTM mapping with high-resolution TF-cistromic data under conditions of specific metabolic manipulation, analysing candidate metabolic pathways that influence TI. Deciphering this interplay will be essential to determine whether metabolic flux is a passive requirement or an active instructor of the ‘poised’ chromatin state that defines innate immune memory.

### DNA methylation and TI

DNA methylation plays a role in regulating immune function, mainly by modulating cell differentiation (Bock et al., 2012). In line with this, monocyte-to-macrophage differentiation relies on DNA demethylation to release the transcriptional silencing of genes related to phagocytosis and macrophage function (Wallner et al., 2016). Moreover, the DNA methylation status also influences macrophage polarisation and immune activation (Wang et al., 2016; Li et al., 2016). In mammals, DNA methylation occurs predominantly at sequences of repeated cytosine and guanine dinucleotides known as CpG islands, which are enriched at gene promoters (Saxonov et al., 2006). The process of DNA methylation involves the transfer of methyl groups from the methyl donor SAM to cytosine by DNA methyltransferases (DNMTs), generating 5-methylcytosine (5mC), which promotes the recruitment of repressor complexes like HDACs, resulting in transcriptional silencing (Klose and Bird, 2006; Nan et al., 1998). De novo methylation is generated by DNMT3A and DNMT3B, while maintenance of methylated DNA, especially during DNA replication, is performed by DNMT1 (Klose and Bird, 2006; Okano et al., 1999). For the process of demethylation, ten-eleven-translocation (TET) proteins act as demethylases by hydroxylating 5mC to generate 5-hydroxymethylcytosine, which is further replaced by cytosine (Guo et al., 2011). Beyond inhibiting histone demethylases by antagonising the cofactor αKG, fumarate and succinate can also affect DNA demethylases, as both enzyme families act through the same mechanism (Figure 1). Nevertheless, the role of DNA methylation/demethylation during TI has not been deeply explored. It was shown that traits of TI elicited by zymosan challenge or *Candida albicans* infection could be transmitted across generations in mice (Katzmarski et al., 2021). This transmission was associated to differential DNA methylation patterns linked to genes important for immune function in the DNA contained within sperm cells of paternal mice (Wallner et al., 2016). Beyond TI, severe infections like sepsis and tuberculosis can trigger a metabolic-epigenetic axis where the accumulation of succinate and itaconate, alongside the nuclear translocation of DNMT3B and TCA enzymes, drives global DNA hypermethylation (Longlax et al., 2024). This process results in the transcriptional silencing of key proinflammatory pathways, establishing a persistent state of immune tolerance (Longlax et al., 2024).

In spite of the understanding of how the interplay between fumarate and αKG can drive TI induction by affecting histone methylation, little is known about the role of SAM, the universal methyl donor for histone and DNA methylation, on TI induction. Early in the innate immune stimulation of macrophages, increases in SAM and succinate/αKG ratios create conditions that promote histone hypermethylation (Seim et al., 2019). It has been reported that 4 hr after β-glucan stimulation, glucose is incorporated into metabolic pathways contributing to OXPHOS, serine biosynthesis, and SAM generation in human monocytes (Cai et al., 2025). A low concentration of lactate, which enhanced trained responses, also increased glucose-derived SAM (Cai et al., 2025). Moreover, methylthioadenosine (MTA), a compound widely used to inhibit TI in vitro (Quintin et al., 2012; Cheng et al., 2014; Moorlag et al., 2020a), is a metabolite produced during SAM metabolism. MTA can inhibit methyltransferase reactions and is structurally similar to S-adenosylhomocysteine, a SAM competitor, highlighting the importance of this metabolite in TI induction. Finally, a study showed accumulation of SAM in alveolar macrophages trained with environmental concentrations of LPS (Zahalka et al., 2022). This evidence highlights the potential contribution of SAM to TI induction and opens the path to investigate SAM biology, as well as the unexplored role of DNA methylation in TI induction.

### Long non-coding RNAs

Epigenetic remodelling can also be regulated by the non-coding part of the genome. Non-coding RNAs (ncRNAs) are a highly heterogenous group of transcripts that are not translated into protein, comprising microRNAs (miRs) and long non-coding RNAs (lncRNAs), among others (Nemeth et al., 2024). MiRs are small RNA molecules (approximately 20–22 nucleotides) that post-transcriptionally control gene expression by targeting mRNAs, in a sequence-dependent manner, causing a block of translation and/or mRNA degradation (Virga et al., 2021b). MiRs can directly target members of the epigenetic machinery affecting the epigenetic state of the cells forming complex epigenetics-miRNA regulatory circuits (Reale et al., 2019; Sato et al., 2011; Virga et al., 2026). As an example, during LPS-induced tolerisation, miR-221 and miR-222 contribute to the reduced expression of several inflammatory genes such as IL-6 and IL-12 by targeting the Brahma-related gene 1 (*Brg1*) in macrophages. The targeting of BRG1 reduces the recruitment of SWI/SNF complex at the promoter region of inflammatory genes, dampening their transcriptional expression (Seeley et al., 2018). Although not described in TI, the well-described role of miRs in epigenetics would suggest that miR can also be involved in the epigenetic circuits that control the trained response.

Besides miRs, lncRNAs are generally defined as ncRNAs larger than 200 nucleotides (Nemeth et al., 2024). Their function is extremely diverse: they can interact directly with the DNA, with other RNA molecules (including miRs) or even with proteins (Nemeth et al., 2024). Recently, a subset of lncRNAs induced by β-glucan, defined as immune gene-priming lncRNAs (IPLs), were linked to the recruitment of chromatin remodelling complexes, acting as scaffold RNAs, to induce H3K4me3 marks (Minton, 2019; Fanucchi et al., 2019). One of the main features of these IPLs is that they are transcribed in close proximity to immune genes within the same chromosome loops, in the same topologically associating domain (TAD), as observed by Hi-C analysis (a technology that combines high-throughput sequencing and ^3^C-chromosome conformation capture – techniques), where they act in *cis* (Fanucchi et al., 2019). These data are in alignment with previous findings showing that co-regulated inflammatory genes are segregated into TADs (Jin et al., 2013). As an example of IPLs, the upstream master lncRNA of the inflammatory cytokine locus (UMLILO) regulates the promoters of chemokine genes such as *Il8*, *Cxcl1*, *Cxcl2,* and *Cxcl3* by recruiting histone methyltransferase complexes resulting in the promotion of H3K4me3 at these selected gene promoters. Molecularly, UMLILO interacts directly with WDR5, an RNA-binding protein that recognises H3K4 methylation, which, in turn, associates with the methyltransferases MLL1 favouring H3K4me3 deposition. In line with this, the deletion of UMLILO abrogates the TNF-induced H3K4me3 mark at the promoters of the selected chemokines, as well as their relative gene transcription. While the majority of the study is carried out in immortalised cell lines, the authors show that the IPLs are induced by NFAT signalling in β-glucan-treated primary monocytes and that NFAT inhibition prevents IPL induction and inflammatory gene expression, suggesting a possible role of IPLs in training. Notably, mouse cells do not express UMLILO in the TAD, where chemokine genes are located and this may explain why β-glucan treatment does not change the induction of chemokines in mouse models and may also contribute to explain why humans are more sensitive to β-glucan-induced inflammatory stimuli compared to mice (Minton, 2019; Gregory et al., 2024).

## Secondary metabolic adaptations that contribute to enhanced immune responses in trained cells

**Figure 2.**
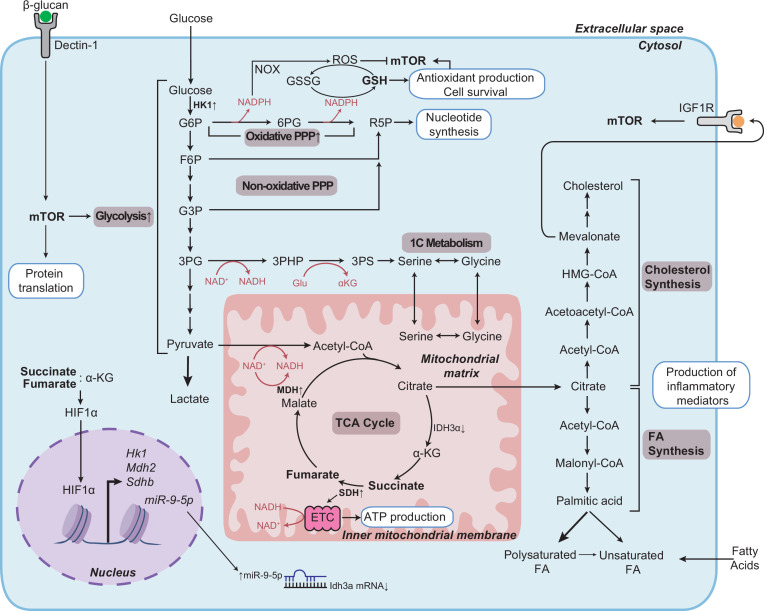
Metabolic adaptations improve immune function in trained cells. Upon myeloid cell detection of training stimuli, such as β-glucan by the receptor Dectin-1, a wide array of metabolic changes take place in the cell. The activation of mTOR is essential for trained immunity (TI) induction. Two of the main effects of this activation are increased protein translation and increased glycolysis. Protein translation can support inflammatory cytokines, as well as components of other cellular processes to enhance cellular function. Glycolysis begins with the conversion of glucose to glucose-6-phosphate (G6P) by the enzyme HK1 and finishes with the generation of pyruvate. G6P can also be utilised in the oxidative PPP to generate R5P required for nucleotide synthesis and NADPH. NADPH is used by NOX to generate ROS. While ROS can be used in several immune responses, in the context of TI, ROS was shown to inhibit mTOR activation and decrease cytokine production. The other role of NADPH is the reduction of glutathione, and subsequent antioxidant function, this allows mTOR activation and promotes cell survival. Further glycolysis metabolites F6P and G3P can also contribute to R5P generation by non-oxidative PPP. The glycolytic metabolite 3PG can be diverted to de novo serine synthesis, a pathway that generates NADH and α-ketoglutarate (αKG), as well as serine and glycine. At the end of glycolysis, pyruvate can be converted to lactate in the cytoplasm, or to acetyl-CoA in the mitochondria to fuel the TCA cycle. In the mitochondria, acetyl-CoA is converted to citrate, which can be exported to the cytoplasm where it fuels FA synthesis and cholesterol synthesis pathways. FAs can also be imported directly into the cytoplasm. Increased intracellular lipid species support the expansion of cell membranes and the production of inflammatory mediators. As well as production of the end product cholesterol, this pathway also generates the intermediate product Mevalonate, which was shown to bind to the receptor IGF1R in an autocrine manner, activating mTOR. Citrate can also be maintained in the mitochondria to continue the TCA cycle. The enzyme IDH3, which results in the generation of αKG, was downregulated by the miRNA miR-9-5p, resulting in decreased αKG levels. This decreases the proportion of αKG to succinate and fumarate, promoting stabilisation of HIF1α, which can then translocate to the nucleus where it acts as a TF to promote glycolysis and inflammatory gene expression. Succinate is converted to fumarate by succinate dehydrogenase (SDH), the expression of which is increased in trained cells. SDH also participates in the ETC as Complex II, contributing to the generation of mitochondrial ATP. The TCA cycle enzyme MDH was also found to be transcriptionally increased, metabolising malate to continue the flow of the TCA cycle. mTOR, mammalian target of Rapamycin; G6P, glucose-6-phosphate; F6P, fructose-6-phosphate; G3P, glyceraldehyde-3-phosphate; 3PG, 3-phosphoglycerate; PPP, pentose phosphate pathway; 6PG, 6-phosphogluconate; R5P, ribose 5-phosphate; NOX, NADPH oxidase; ROS, reactive oxygen species; GSSG, glutathione disulfide; GSH, glutathione; NADPH, nicotinamide adenine dinucleotide phosphate (reduced form); NADP^+^, nicotinamide adenine dinucleotide phosphate (oxidized form); NADH, nicotinamide adenine dinucleotide (reduced form); NAD^+^, nicotinamide adenine dinucleotide (oxidized form); 3PHP, 3-phosphohydroxypyruvate; 3PS, 3-phosphoserine; IGF1R, insulin-like growth factor 1 receptor; TCA, tricarboxylic acid; MDH, malate dehydrogenase; SDH, succinate dehydrogenase; IDH3α, isocitrate dehydrogenase (NAD(+)) 3 catalytic subunit alpha; Glu, glutamate; αKG, alpha ketoglutarate; FA, fatty acid; CoA, coenzyme A; ETC, electron transport chain; ATP, adenosine triphosphate; HIF1a, hypoxia inducible factor 1 subunit alpha, mRNA, messenger RNA; miRNA, micro RNA.

While epigenetic changes in trained macrophages have been directly linked to increased cytokine induction, transcriptional regulation of metabolic pathways or changes in their activation could also indirectly allow superior immune responses in these cells due to increased availability of nucleotides, proteins, and lipids required to mount an immune response. Macrophages are known to be metabolically flexible cells, and this allows them to function in a wide range of physiological niches (Wculek et al., 2022). Upon activation by cytokines or PAMPs, macrophages increase their bioenergetic demands. While the epigenetic consequences of training are discussed above, we will now focus on the metabolic consequences that occur post-transcription that can affect the secondary response. Such adaptations provide the resources required for mRNA transcription, protein synthesis, membrane biogenesis, and redox homeostasis, thereby supporting prolonged and enhanced cytokine production that underlies a trained immune response (Figure 2). Further studies are required to investigate the role of distinct metabolic pathways in the TI response. Here, we aim to summarise what studies have revealed so far about the role of metabolism in supporting the TI phenotype.

### Glycolysis and OXPHOS

There is much evidence supporting the influence of metabolic intermediates on epigenetic changes (Fanucchi et al., 2021); however, this relationship is not unidirectional. There is also evidence that epigenetic changes can lead to metabolic reprogramming, and this can have effects that are more wide-ranging than transcriptional regulation. Common to all training stimuli is the induction of glycolysis. A landmark study showed that β-glucan-trained monocytes increase glycolysis via an Akt-mTOR-HIF axis (Cheng et al., 2014). In these trained monocytes on day 7, glucose consumption, lactate production, and the NAD^+^/NADH ratio were increased (Cheng et al., 2014). β-Glucan-trained monocytes show increased H3K4me3 and H3K27ac histone marks in key glycolysis genes, as well as genes of the mTOR pathway, upon β-glucan training (Cheng et al., 2014). Similarly, in BCG-induced TI, an increase in glycolysis, as well as glutaminolysis, was reported, with a concurrent increase in H3K4me3 marks and a decrease in H3K9me3 marks at promoters for glycolysis pathway genes and mTOR (Arts et al., 2016a). Glycolysis is regulated by a series of enzymes that can be transcriptionally regulated to increase enzyme expression, allowing an increase in glycolytic capacity in cells. In line with the epigenetic changes in glycolysis genes, there was an increase in glucose consumption and lactate production in β-glucan-trained cells, indicating a functional increase in glycolysis (Cheng et al., 2014). Activation of mTOR has been shown to regulate HIF1α stability, permitting increased binding to HIF1α target genes, such as glycolytic enzymes (Land and Tee, 2007). Overexpression of Glut1, the main glucose transporter in macrophages, was shown to be sufficient to enhance inflammatory cytokine production in vitro via ROS induction (Freemerman et al., 2014). The relevance of this in vivo was observed when bone marrow from hyperglycaemic mice was found to result in BMDMs with higher inflammatory gene induction in response to LPS and IFNγ, indicating hyperglycaemia-induced TI (Edgar et al., 2021). This hyperglycaemia-induced memory was associated with higher global H3K4me3 and H3K27ac marks, in both in vitro systems and aortic root plaque macrophages, promoting more plaque formation in a model of atherosclerosis (Edgar et al., 2021). While the focus of these studies is gene regulation, glycolysis has many more wide-ranging effects with the potential to enhance immune responses, such as an increase in TCA cycle metabolites, ROS, and promoting FAS to name a few (Soto-Heredero et al., 2020). Further analysis of these downstream effects of catabolic metabolism would shed light on the functional role of this increase in glycolysis in TI.

While trained cells were initially thought to decrease OXPHOS in favour of glycolysis, recent results support the conclusion that both glycolysis and OXPHOS are maintained in trained cells before secondary stimulation (Cheng et al., 2014; Arts et al., 2016a; Keating et al., 2020b). While ‘oxidative phosphorylation’ was the most upregulated pathway upon β-glucan stimulation in macrophages, the effect of this transcriptional regulation remains unclear (Arts et al., 2016b). Administration of oligomycin had no effect on cytokine production in trained cells, indicating that mitochondrial ATP is not required for TI induction (Arts et al., 2016a). A subsequent study showed an increase in H3K4me1 marks at enhancers of TCA cycle enzymes MDH2 and SDHB was partially dependent on the lysine methyltransferase Set7 (Keating et al., 2020b). Specifically, these marks correlated with increased Mdh2 and Sdhb expression, an increase in TCA cycle metabolites such as fumarate and malate, and an increase in mitochondrial oxygen consumption (Keating et al., 2020b). Similarly, in human monocytes, epigenetic changes were detected as early as 1 hr after β-glucan stimulation at genes involved in glutathione (GSH) metabolism, TCA cycle, and the PPP (Ramendra et al., 2021). These changes correlate with enhanced immune responses, yet whether this transcriptional regulation of metabolic genes is essential for TI is difficult to ascertain. Therefore, the contribution of OXPHOS to metabolite regulation (such as succinate accumulation), redox balance (NAD^+^/NADH and FAD/FADH_2_), membrane potential, and the ATP/ADP balance remains to be explored.

### ROS and redox balance

The PPP utilises the glycolysis intermediate glucose-6-phosphate (G6P) to generate NADPH for ROS production, lipid synthesis, and redox balance via the generation of reduced GSH, as well as ribose-5-phosphate for nucleotide synthesis (Figure 2). In the case of oxLDL-induced TI, an mTOR-dependent increase in cellular ROS was detected (Sohrabi et al., 2019). Following β-glucan or BCG stimulation during TI induction, both intracellular ROS levels and antioxidant gene expression were increased (Ferreira et al., 2021). In monocytes stimulated with β-glucan, it was found that the reduced form of GSH increased over the course of the training protocol, whereas no change in the oxidised form, GSSG, was detected, indicating an enhanced cellular antioxidant capacity in trained cells (Su et al., 2021a). While genes involved in GSH metabolism correlated with TI features in human monocytes, pharmacologically decreasing GSH levels had mild effects on secondary cytokine production (Ferreira et al., 2021). However, a genetic approach targeting the catalytic subunit of glutamate-cysteine ligase (Gclc) showed impaired GSH synthesis and subsequently decreased activation of mTOR, glycolysis, glutaminolysis, and decreased responsiveness upon β-glucan training in vitro and in vivo (Su et al., 2021a). Gclc deletion resulted in increased ROS levels that inhibited mTOR function and could be rescued with treatment with the antioxidant NAC (Su et al., 2021a). This data points to ROS as a negative regulator of TI and the necessity of oxidative stress management in these cells. In line with this, in human monocyte, GSH was shown to be essential for cell survival after β-glucan stimulation, indicating increased oxidative stress in trained myeloid cells that requires a strong antioxidant metabolism to compensate promote cell survival and function ([Bibr bib118][Bibr bib118]).

Interestingly, despite the importance of GSH regulation, the use of the PPP inhibitor 6-aminonicotinamide was shown to have no effect on BCG-induced monocyte training (Arts et al., 2016a). Another glucose-derived biosynthetic pathway that can support redox balance is the de novo serine synthesis pathway. This pathway utilises the glycolysis intermediate 3-phosphoglycerate to catalyse the production of serine to support one-carbon metabolism, as well as protein translation (Figure 2). In the context of primary LPS stimulation, this pathway was shown to support proinflammatory cytokine production via GSH synthesis and redox balance maintenance (Rodriguez et al., 2019). Notably, LPS treatment of macrophages/monocytes, at concentrations typically associated with the induction of tolerance (Ifrim et al., 2014; Sly et al., 2004), has been shown to increase ROS production (Hsu and Wen, 2002; Casey et al., 2025) and increase the GSH/GSSG ratio similarly to β-glucan. However, while β-glucan-trained cells display an increase in total GSH levels, LPS stimulation showed no change compared to unstimulated cells (Ramendra et al., 2021). It is possible that changes in GSH levels are one factor that could help explain the non-monotonic dose-response of LPS.

### Lipid regulation

Lipids are key to macrophage inflammatory function (Yan and Horng, 2020). Monocytes exposed to β-glucan were found to differentiate into macrophages with elevated expression of cholesterol synthesis pathways (Saeed et al., 2014). A more in-depth study of metabolic changes over time comparing primary stimulation with β-glucan or LPS showed upregulation of TCA metabolites, as well as increased fatty acid and cholesterol synthesis 6 days after β-glucan treatment (Arts et al., 2016b). A transcriptional increase in cholesterol biosynthesis was also demonstrated in bone marrow progenitors exposed to β-glucan in vivo. Lipidomic analysis of these progenitors from β-glucan-trained mice showed accumulation of short-chain lipids with saturated acyl chains and cholesterol esters, whereas control cells contained more polyunsaturated fatty acids and lipids with longer acyl chains. These changes in trained mice were associated with the skewing of hematopoietic progenitors towards the myeloid lineage (Mitroulis et al., 2018). This finding aligns with epigenetic analysis of multipotent progenitors (MPPs), which are early hematopoietic subsets responsible for generating mature immune cells. When sorted from mice trained with MV130, these MPPs displayed an increase in chromatin accessibility around genes involved in cholesterol homeostasis, amongst others (Brandi et al., 2022).

Cholesterol is essential for membrane integrity and the production of inflammatory mediators. Its synthesis is mediated by the mevalonate pathway, which is subject to tight transcriptional and post-transcriptional regulation by AKT and mTOR signalling (Lee and Bensinger, 2022). Inhibition of cholesterol synthesis with statins was found to dampen TI induction (Arts et al., 2016b). In line with this association, single-cell transcriptomic analysis of CD45^+^ cells in the human carotid artery showed that lipid accumulation in plaque macrophages was linked to increased inflammation (Dib et al., 2023). However, despite the link between cellular cholesterol and cytokine production, further studies into the role of cholesterol synthesis in TI revealed that the importance of this pathway was not due to cholesterol synthesis per se, but rather the intermediate mevalonate (Bekkering et al., 2018). Indeed, mevalonate addition alone promoted a trained phenotype in monocytes, via binding to the insulin-like growth factor 1 receptor (IGF1R), leading to a subsequent activation of mTOR and glycolysis induction (Bekkering et al., 2018; Figure 2). This study implicates metabolites induced downstream of training stimuli in a feedforward loop to induce further trained responses. Interestingly, IGF1R expression is much more ubiquitous than the expression of PRRs found on professional immune cells, opening the question of whether training can be propagated to non-immune cell types by metabolite communication.

As well as cholesterol synthesis, FAS is also upregulated in trained macrophages (Arts et al., 2016b). Saturated fatty acids have shown the ability to act as PAMPs, inducing inflammatory immune cell activation in various disease contexts (Christ and Latz, 2019). In line with this finding, palmitic acid was shown to synergise with LPS to induce high levels of intracellular ceramide production, promoting increased inflammatory cytokine production in macrophages (Schilling et al., 2013). The effect both on ceramide synthesis and cytokine production could be reverted by incubation of cells with the mono-unsaturated oleic acid (Seufert et al., 2022). In the context of a ketogenic diet, which is high in saturated fatty acids, palmitic acid was found to induce a hyperinflammatory response to subsequent LPS challenge in macrophages (Seufert et al., 2022). While saturated fatty acids appear to act directly as inflammatory mediators, in the case of canonical training stimuli such as BCG with physiological fatty acid levels, it was shown that the synthesis of long-chain polyunsaturated fatty acids and the subsequent generation of lipid mediators was essential for the induction of trained immune responses (Ferreira et al., 2023a). Therefore, the role of fatty acids in TI depends on their structure, contributing either as immune agonists or metabolic regulators.

The bioavailability of substrates for these metabolic processes is strongly influenced by diet. There are many studies linking a ‘Western-like’ diet, as well as ultra-processed food consumption with more inflammatory biomarkers; however, most of these studies do not separate primary immune responses from secondary stimulation and the existence of immune memory (Barbaresko et al., 2013; Maki et al., 2024). Since the definition of TI, it has now been established that oxidised LDL alone acts as a training stimulus in monocytes (Bekkering et al., 2014; Keating et al., 2020a; Christ et al., 2018). This effect could be recapitulated in vivo with a Western diet, which induces hyperlipidaemia and hypercholesterolaemia, promoting TI hallmarks in monocytes and bone marrow progenitors (Christ et al., 2018). This finding is in line with previous studies showing that the Abca1 and Abcg1 cholesterol efflux receptors suppress myelopoiesis (Yvan-Charvet et al., 2010), indicating a clear link between intracellular lipid levels and a trained phenotype. The TI reprogramming observed in the context of Western diet was due to activation of the NLRP3 inflammasome (Christ et al., 2018). OxLDL and cholesterol have previously been shown to be converted to NLRP3-activating crystals in a CD36-dependent mechanism to promote IL-1β production (Sheedy et al., 2013). This indicates a direct immune agonist function of lipids as well as their metabolic ability to support immune function. However, not only direct dietary lipids affect TI. In a study of rural and urban populations in Tanzania, it was found that rural populations consuming a largely plant-based diet showed an increase in food-derived flavones in plasma, whereas urban populations displayed an increase in primary bile acid and cholesterol metabolism pathways (Temba et al., 2021). One highly expressed plasma flavone, Apigenin, was found to reduce primary inflammatory cytokine production (Temba et al., 2021). As well as direct effects described for these nutritional components remains their indirect effect via the microbiota, with fibre-rich diets supporting a diverse microbiota capable of producing high levels of short chain fatty acids known for their immunomodulatory effects, whereas high-fat diets support secondary bile acid generation and intestinal permeability known to promote inflammation (Rohr et al., 2020; Zeng et al., 2019). Certain translocated bacterial species have also been shown to directly induce TI in mouse models (Robles-Vera et al., 2025). Therefore, dietary associations with TI are very complex, with both direct and indirect associations to increased immune cell priming as well as primary immune responses reported. What appears clear from these studies is that Western diets promote TI and an increase in inflammatory phenotypes through a range of mechanisms. The metabolic consequences of each of these mechanisms require further study.

### Post-transcriptional regulation

Cell metabolism can also be regulated by post-transcriptional mechanisms. For example, miRs affect cell metabolism by directly targeting metabolic enzymes or transporters, as well as by controlling pathways involved in the control of metabolic processes, such as mTOR signalling (Virga et al., 2021b; Carrà et al., 2024). Different metabolic pathways, including glycolysis, the TCA cycle, OXPHOS, lipid, and amino acid metabolism, have been shown to be controlled by miRs such as miR-210 (Virga et al., 2021a), miR-146a (Runtsch et al., 2019), miR-33 (Ouimet et al., 2015), among others, in a cell-specific and context-dependent manner (Virga et al., 2021b; Carrà et al., 2024). Notably, miRs are regulated by training stimuli, such as β-glucan, suggesting that they might be involved in TI. In the monocyte-like THP-1 cell line, β-glucan induces the expression of miR-193-5p, miR-5787, miR-146a, miR-210-3p, miR-30a-5p, and miR-8072 (Du et al., 2017), while in the macrophage-like Raw264.7 cell line, β-glucan induces miR-32-5p (Yang et al., 2025). Similarly, miRs expression profiles of β-glucan-trained peripheral blood mononuclear cells from human and mouse specimens show distinct pattern of differentially expressed miRs upon training. Specifically, miR-9-5p, let-7i-5p, miR-223-3p, and miR-155 are consistently upregulated while miR-152-3p, miR-328-3p, and miR-29a-5p are consistently downregulated upon training in both human and mouse (Su et al., 2021b). An in-depth study of role of miR-9-5p, one of the miR consistently upregulated in both species upon training, shows that its depletion reduces training phenotype both in vitro and in vivo and reduces mice survival during *C. albicans* infection. This evidence provides the first *proof-of-concept* of the functional relevance of miRs in TI (Su et al., 2021b). Molecularly, miR-9-5p directly targets IDH3α (isocitrate dehydrogenase 3α), an enzyme of the TCA cycle involved in the oxidative decarboxylation of isocitrate to αKG, reducing αKG levels, stabilising HIF-1α and promoting the glycolytic switch upon training (Figure 2). Conversely, the depletion of miR-9-5p in trained macrophages widely affects cell metabolism by reducing glucose consumption, glycolysis, and NAD, while increasing the ratio of αKG to succinate and fumarate. Due to this increased αKG/fumarate ratio, depletion of miR-9-5p in trained macrophage enhances the activity of the demethylases KDM5, an enzyme involved in the removal of lysine trimethylation of H3K4me3, influencing the methylation status at the promoter of inflammatory and metabolic genes such as *Tnf*, *Il6*, *Hk2*, and *Slc2a1 (Glut1*) limiting the glycolytic switch and enhanced cytokine expression.

Another interesting mechanism of post-transcriptional regulation is provided by the essential glycolysis component GAPDH. In monocytes, it has been described that GAPDH protein can bind to the 3’ untranslated region (UTR) of several mRNA species, such as *Tnf*, to induce post-transcriptional repression (Millet et al., 2016). Notably, this mechanism had previously been shown in T cells in the regulation of *Ifng* mRNA (Chang et al., 2013). Therefore, although not investigated in training setting, the increased glycolysis may also increase TNF levels via the occupation of GAPDH with glycolytic machinery, preventing its secondary inhibitor function. In line with the potential importance of post-transcriptional regulation, inhibiting the glycolytic burst observed upon TLR stimulation in dendritic cells was shown to have no effect on cytokine transcription, but to inhibit FAS required for the increased protein synthesis and cytokine export (Everts et al., 2014). As well as regulating glycolysis, mTOR is a master regulator of many biological functions, including protein translation and mitochondrial activity (Morita et al., 2013). Given the importance of mTOR in the induction of TI, its role may be multifaceted, extending beyond glycolysis induction and HIF stabilisation (Cheng et al., 2014).

### Metabolic stress

The concept of TI highly resembles the concept of transcriptional memory in which external stimulus can prime a cellular state that results in altered expression of primed genes upon re-exposure to inducing signals (Tehrani et al., 2025). It is characterised by transient gene activation leading to a primed state maintained in the absence of active transcription (Tehrani et al., 2025). In other words, stimuli trigger behaviours, a complex process of mechanisms and features that are employed by living organisms to adapt to the constantly changing environment. When applied to the innate immune system and its ability to react to subsequent pathogenic challenges, this process can be called TI. Transcriptional memory occurs in a diversity of organisms, including both prokaryotes and eukaryotes. Nutrients and stress conditions are common triggers of this phenomenon (Tehrani et al., 2025). An illustrative example is found in plants, in which dehydration, infection, high salt, and temperature shock induce primed transcriptional states to control future stressors (Fu and Dong, 2013; Ding et al., 2012; Lämke et al., 2016). TFs are important contributors to transcriptional and epigenetic memory and they cooperate with local chromatin-based mechanisms like histone-modifying enzymes to induce memory (Tehrani et al., 2025). In line with this, stress-mediated TFs are associated with TI induction. Interestingly, ATF factors were reported in several works regarding TI and tolerance induction. For instance, ATF7 phosphorylation and subsequent release from chromatin was found to be involved in innate immune memory, by decreasing repressive histone marks (Yoshida et al., 2015). ATF4 TF motifs are enriched in the accessible regions generated upon TI mediated by low-dose LPS, compared with high-dose LPS that induces tolerance (Zhang et al., 2022). Accordingly, chronic psychological stress led to a heightened state of readiness in the innate immune system, characterised by increased production of proinflammatory cytokines and an enhanced response to secondary immune challenges (Barrett et al., 2021). Moreover, regarding long-term memory traits, it was found that cardiac stress produced after heart failure influenced the epigenetic landscape of hematopoietic stem cells, thereby affecting their ability to produce different cardiac macrophage subpopulations with enhanced proinflammatory activity. This highlights the potential implications of metabolic stress in TI induction and the contribution of the stress response and adaptations to the epigenetic rewiring (Rettkowski et al., 2025).

## Technical approaches to study the metabolic rewiring in TI

Until now, most analyses in TI are performed at a single timepoint, or at a limited set of time intervals at best. As discussed earlier, it is important to discriminate between early and late metabolic changes in TI and we would therefore advocate for more extensive kinetic profiling of the epigenetic and metabolic state of myeloid cells in future studies. Currently, the technologies that are applied to profile metabolic and subsequent epigenetic changes in TI range from very simple multi-well-plate-based assays to high-end OMICs analyses. To investigate epigenetic changes in response to metabolic reprogramming, western blotting can be performed using antibodies directed against histone modifications of interest. This approach has the capacity to detect broad epigenetic changes but doesn’t provide insight into the epigenetic landscape of regulatory region of specific (inflammatory) genes. To obtain such information, one should perform chromatin immunoprecipitation (ChIP) with antibodies targeting specific epigenetic marks of interest. Next, a quantitative PCR can be performed with specific primer pairs to compare DNA fragment abundance in precipitate versus non-precipitated (input) samples. A more sophisticated unbiased approach is to sequence the immunoprecipitated chromatin (ChIP-seq). These ChIP-seq experiments are frequently accompanied by ATAC-seq and RNA-seq to obtain complementary information about specific epigenetic marks, chromatin accessibility, and the expression of the associated genes. ChIP-based studies come with the difficulty that they rely on good antibody clones, and that protocols are not fully standardised and may require trial-and-error-based optimisations that complicate progress and comparison with the field. Also, it remains difficult to probe the relative abundance, and hence importance of, the multitude of histone modifications that can occur and how they influence each other, and eventually how they regulate TI.

Both ATAC-seq and RNA-seq are becoming regularly used at the single-cell level and together with the emergence of cell-based metabolic techniques, it is clear that TI research is making the transition towards investigations at single-cell resolution. Another apparent evolution in the field is the study of epigenetic architectures and long-range contact between regulatory regions in the genome. For example, Ziogas et al. recently showed how glycolysis-derived H3K18la marks affect long-range contacts in the regulatory regions of inflammatory genes like *Il1b*, thus identifying a role for metabolic rewiring and subsequent histone lactylation in modifying gene architecture (Ziogas et al., 2025).

Moreover, it should be emphasised that most current techniques are still largely candidate-based targeted approaches that come with the limitation that they provide a biased and non-comprehensive view. Newer mass spectrometry (MS)-based characterisation of PTMs can reveal a more complete and quantitative analysis of histone marks in TI (Noberini et al., 2022; Thomas et al., 2020). While MS can reveal the existing modifications and their relative abundance in great detail, it does not provide information about their position. As such, a combination of complementary techniques will be needed to uncover a complete picture containing unbiased quantitative information about histone marks and their relative contributions (MS), distribution of specific marks across the genome (ChIP-seq), how this affects DNA accessibility (ATAC-seq), and finally gene transcription (RNA-seq).

For immunometabolic profiling, commercial colourimetric or fluorimetric assay kits can be used to measure intracellular and extracellular levels of lactate, acetyl-CoA, and other key metabolites that govern epigenetic modifications in TI. A broader range of metabolites can be assessed using MS-based targeted and untargeted metabolomics. Importantly, those bulk techniques do not reveal the localisation of metabolites across different organelles in the cell. Subcellular fractionation, followed by metabolite measurements, can be performed to obtain such information. This is relevant since multiple metabolic enzymes that generate metabolites needed for histone modifications have been shown to travel to the nucleus where they can increase concentrations at the location where they are consumed by histone-modifying enzymes (Lazaropoulos et al., 2024; Shao et al., 2024).

While metabolomics data can provide a snapshot of metabolite abundance at a certain timepoint, applying ^13^C stable isotopes enables the quantification of intracellular fluxes through diverse metabolic pathways and is often termed fluxomics or ^13^C metabolic flux analysis. Most of the metabolic readouts applied in TI are bulk analyses and it should be noted that novel approaches that interrogate metabolism at the single-cell level are becoming more regularly applied in the immunometabolism field (Artyomov and Van den Bossche, 2020; Cosgrove et al., 2024). These include cytometry-based approaches like ‘Met-Flow’ (Ahl et al., 2020) and single-cell metabolic profiling (scMEP Hartmann et al., 2021) via CyTOF in which fluorescently labelled or heavy-metal-tagged antibodies against key transporters and rate-limiting enzymes across multiple metabolic pathways are applied to estimate the metabolic configuration of immune cells at a per-cell level. So far, both Met-Flow and scMEP haven’t been applied in the TI context, but the recent use of SCENITH and CENCAT approaches indicate the field is moving from bulk towards single-cell metabolic analyses.

Aside from analysis of expression levels, there also exist functional readouts of metabolic activity. The most commonly used of these is the Seahorse extracellular flux analysis. This technique is commonly used in immunometabolism and TI research to measure glycolytic and mitochondrial metabolism via real-time measurements of protons and oxygen to calculate the extracellular acidification rate and oxygen consumption rate, respectively. However, this analysis requires a certain cell number which can be difficult to achieve in scarce populations and does not give single-cell data. A complementary single-cell approach is SCENITH – a flow cytometry-based methodology that uses puromycin incorporation as a proxy for protein synthesis, a process that is responsible for a large proportion of total ATP usage by a cell (Argüello et al., 2020). Using the glycolysis inhibitor 2-deoxyglucose and mitochondrial ATP synthase inhibitor oligomycin and assessing their effect on puromycin incorporation, one can thus calculate the glycolytic versus mitochondrial dependencies of immune cell subsets. This approach was recently applied to explore metabolic changes in ‘trained’ γδ T cells upon measles, mumps, rubella (MMR) vaccination (Röring et al., 2024). CENCAT is an alternate click chemistry-based measurement of protein synthesis via biorthogonal noncanonical amino acid tagging to profile the metabolic dependencies of cell subsets (Vrieling et al., 2024). This new method was recently applied to measure the bioenergetic profile of BCG-trained versus LPS-tolerised macrophages (Ziogas et al., 2025), albeit at a bulk level. The power of the cytometry-based metabolic profiling approaches is that they allow the assessment of metabolic and immune phenotypes simultaneously and a single-cell or subset resolution. They thus have the potential to address key outstanding questions in the field by uncovering how specific monocyte subsets and phenotypes are ‘trained’ and differentiate into macrophages.

### Conclusion

There is now a large body of evidence that TI is the result of a coordinated epigenetic and metabolic rewiring. Many of the metabolic pathways mentioned here are not specific to training stimuli. Indeed, LPS, the canonical driver of endotoxin tolerance and immune paralysis, is well known for its activation of mTOR, glycolysis, and HIF stabilisation, as well as cytokine and growth factor receptor signalling (Tannahill et al., 2013; Covarrubias et al., 2015; Borriello et al., 2017). Whether these metabolic pathways support all macrophage activation and the difference lies in epigenetics, or whether the exact combination of metabolic pathways upon training stimuli is crucial for enhanced long-lasting responsiveness to activation, remains to be deciphered. Although a wide range of epigenetic modifications have been described in the context of TI, their possible interconnections and their relative contributions to TI remain poorly defined, and some epigenetic modifications have not yet been studied in this context (Baldensperger and Glomb, 2021; Xie et al., 2016). While there are many unanswered questions in this field, with each new discovery, the common factors become clearer. With the increasing use of new state-of-the-art technologies, these answers become more within reach, and their importance in human disease can be exploited.
